# Free-water diffusion tensor imaging improves the accuracy and sensitivity of white matter analysis in Alzheimer’s disease

**DOI:** 10.1038/s41598-021-86505-7

**Published:** 2021-03-26

**Authors:** Maurizio Bergamino, Ryan R. Walsh, Ashley M. Stokes

**Affiliations:** 1 Division of Neuroimaging Research, Barrow Neurological Institute, Phoenix, AZ 85013 USA; 2 Muhammad Ali Parkinson Center, Barrow Neurological Institute, Phoenix, AZ 85013 USA

**Keywords:** Dementia, Neurodegenerative diseases, Biomarkers

## Abstract

Magnetic resonance imaging (MRI) based diffusion tensor imaging (DTI) can assess white matter (WM) integrity through several metrics, such as fractional anisotropy (FA), axial/radial diffusivities (AxD/RD), and mode of anisotropy (MA). Standard DTI is susceptible to the effects of extracellular free water (FW), which can be removed using an advanced free-water DTI (FW-DTI) model. The purpose of this study was to compare standard and FW-DTI metrics in the context of Alzheimer’s disease (AD). Data were obtained from the Open Access Series of Imaging Studies (OASIS-3) database and included both healthy controls (HC) and mild-to-moderate AD. With both standard and FW-DTI, decreased FA was found in AD, mainly in the corpus callosum and fornix, consistent with neurodegenerative mechanisms. Widespread higher AxD and RD were observed with standard DTI; however, the FW index, indicative of AD-associated neurodegeneration, was significantly elevated in these regions in AD, highlighting the potential impact of free water contributions on standard DTI in neurodegenerative pathologies. Using FW-DTI, improved consistency was observed in FA, AxD, and RD, and the complementary FW index was higher in the AD group as expected. With both standard and FW-DTI, higher values of MA coupled with higher values of FA in AD were found in the anterior thalamic radiation and cortico-spinal tract, most likely arising from a loss of crossing fibers. In conclusion, FW-DTI better reflects the underlying pathology of AD and improves the accuracy of DTI metrics related to WM integrity in Alzheimer’s disease.

## Introduction

Alzheimer’s disease (AD) is a neurodegenerative disorder and the most common cause of dementia in older adults^1^. Current biomarkers for AD target the dominant pathological paradigm, characterized by beta-amyloid and tau pathologies, as well as neurodegenerative changes. While much of the pathologic evidence of AD is found in gray matter, it is well recognized that concomitant white matter (WM) alterations occur in the brains of AD populations, suggesting an expanded role for WM-based biomarkers in AD^2^.

Diffusion-based magnetic resonance imaging (dMRI) enables the in vivo evaluation of brain microstructure and, more specifically, WM integrity by probing water diffusivity associated with axons^3^; most commonly, diffusion-related parameters are obtained by analysis using the diffusion tensor imaging (DTI) model. In WM, faster diffusivity along the fibers, with lower perpendicular diffusivity, yields a higher fractional anisotropy (FA). While decreased FA has been associated with demyelination, edema, gliosis, and inflammation, FA is unfortunately an indirect marker of WM integrity because the individual microscopic contributions of various non-WM components cannot be disentangled^4,5^. Other DTI-derived metrics include axial diffusivity (AxD), which may be related to axonal damage, and radial diffusivity (RD), which is correlated with myelin integrity, axonal diameter and density, and fiber coherence^6^. Prior DTI studies have shown that AD is associated with lower FA and higher AxD and RD values across multiple regions in the WM, including the corpus callosum (CC), fornix, cingulum, inferior longitudinal fasciculus (ILF), and superior longitudinal fasciculus (SLF)^7–9^. Moreover, changes in DTI metrics may precede both neuronal loss and symptoms onset, suggesting their potential role as early biomarkers. While several studies have shown a correlation between DTI-derived metrics and cognitive scores^10–12^, other similar investigations have failed to show a significant correlation^13–16^ suggesting methodologic and/or analytic impacts on dMRI-derived data.

Although dMRI is a well-recognized method for assessing WM, it has several limitations, including known inaccuracies related to partial volume effects (PVEs), wherein the resulting DTI metrics reflect a weighted average of multiple diffusion components within a voxel and are thus no longer specific to a single tissue type^17^. The impact of PVEs may be particularly profound in AD, where neuronal loss leads to secondary *ex vacuo* increases in cerebrospinal fluid (CSF) and free water (FW) across the brain. To overcome the effects of extracellular FW on DTI-derived metrics, a FW correction algorithm has been developed to quantify and remove the contribution of extracellular FW^18^. FW-DTI has been shown to improve both tissue specificity^19^ and DTI-based tract reconstruction^18^. This model has previously been used to study several neurological diseases, including AD^20–24^, Parkinson’s disease^25^, and depression^26,27^.

Using FW-DTI in a healthy aging population enriched for AD risk, Hoy et al. found that DTI-based markers of altered microstructure were significantly associated with CSF biomarkers of preclinical AD pathology^20^. FW correction may be particularly important in the fornix, due to both its proximity to the lateral ventricles and its association with the hippocampus^22^, and may also improve sensitivity to conversion to mild cognitive impairment (MCI) relative to uncorrected DTI metrics^23^. The FW index has separately been proposed as a standalone biomarker of AD-related pathology. This index has been associated with cognitive decline in multiple domains and may enable separation of vascular and neurodegenerative contributions to cognitive decline in the presence of mixed dementia^21^. More recently, an elevated FW index was observed in MCI and AD patients compared with healthy controls (HC), even after correction for white matter hyperintensities^28^. However, none of these studies have directly compared standard and FW-DTI metrics in the context of distinguishing between AD and HC. As AD-related WM degeneration leads to a significant intravoxel FW component, FW-DTI may improve sensitivity to early brain changes and provide a clearer and more specific interpretation of results compared to standard DTI.

In this study, we compared FA, AxD, RD, and MA from both standard DTI and FW-DTI between AD and HC subjects obtained from the Open Access Series of Imaging Studies (OASIS-3) brain project^29^. This dataset was previously used to investigate AD-related changes in the size and shape of the CC^30,31^. We also investigated the complementary FW index for both groups and analyzed voxel-based correlations between all DTI-derived metrics (from DTI and FW-DTI) and the mini-mental state examination (MMSE) score^32^. We hypothesize that FW-DTI will improve the sensitivity and specificity for detection of WM tract abnormalities in AD. More specifically, increased FW due to neurodegeneration reduces the accuracy of diffusion-derived metrics, and thus, the removal of FW contributions improves the estimation of DTI metrics and may increase confidence in the patterns observed across DTI studies. In addition, the ability to quantify FW may yield complementary insight into sub-voxel WM neurodegenerative processes. The application of FW-DTI for AD better reflects the underlying pathology and should thus further improve interpretation of AD WM pathology.

## Materials and methods

### Subjects

All data were downloaded from the OASIS-3 brain project (http://oasis-brains.org/), which is a neuroimaging dataset for normal aging and Alzheimer’s disease and is freely available to the scientific community. Inclusion criteria were participants between the ages of 60 and 90 years who had 3 T dMRI data available with more than 30 directions. Exclusion criteria were cognitively impaired participants with dementia not attributed to AD, healthy control participants with significant comorbidities, and participants for whom the MMSE was not available. Only one time-point was used per subject. Subsequently, this study included 30 HCs (17 females; age (standard deviation, S.D.) = 73 (6) years) and 28 AD subjects (primarily mild AD; 15 females; age = 75 (7) years). Four AD subjects had remote mood disorders and one had a remote hypothyroidism as secondary medical diagnoses. The Clinical Dementia Rating (CDR)^33^, which is a 5-point scale used to characterize multiple domains of cognitive and functional abilities, was zero for all HCs and between 0.5 and 3.0 for the AD group. The MMSE is a 30-point assessment commonly used to measure cognitive impairment. The mean value of MMSE was 29.10 (S.D. 1.24) for HC and 24.18 (S.D. 5.02) for AD. All subject characteristics are summarized in Table 1.

**Table 1:** Group demographics with MMSE and CDR scores.

	N subjects	N females	Age	MMSE	CDR
HC	30	17	73 (6)	29.10 (1.24)	0 (0)
AD	28	15	75 (7)	24.18 (5.02)	0.82 (0.55)
*t-test*			t = − 1.51; *p* = 0.137	t = − 5.21; *p* < 0.0001*	t = − 8.22; *p* < 0.0001*

CDR		HC		AD	
CDR 0 (none)		30		0	
CDR 0.5 (very mild)		0		16	
CDR 1 (mild)		0		10	
CDR 2 (moderate)		0		1	
CDR 3 (severe)		0		1	

*Student t-test demonstrated differences in MMSE and CDR between AD and HC cohorts.

### MRI protocol for DTI

All dMRI data collected from the OASIS-3 database were acquired using a 3 T scanner (Siemens). For all subjects, the acquisition was performed using 64 diffusion-encoding directions (b value: 1000 s/mm^2^; TR/TE: 11,000/87.0 ms; flip-angle = 90°; matrix: 96 × 96; field of view (FOV): 24.0 × 24.0 cm; slice thickness: 2.5 mm; 64 axial slices) and one non-diffusion-weighted image (B0 image).

### Data preprocessing

DMRI data were downloaded in NIFTI format and processed as previously described^26,27^. Briefly, the data were preprocessed using the functional magnetic resonance imaging of the brain (FMRIB) software library tool (FSL, version 5.0.4)^34^. Following translation and rotation estimation, the raw dMRI images were corrected for eddy currents and motion^35^. After motion correction, the gradient orientations were compensated (prior to calculating B-matrices) to account for the rotational component of registration. A brain mask was defined for each subject using the Brain Extraction Toolbox (BET)^36^ for the single B0 images. DTI standard (uncorrected for FW) and FW-corrected maps were calculated using an in-house MATLAB script^26^, which is available upon request. The FW maps were computed by fitting the following model at each voxel^18^:

1 $${A}_{g}\left(D,f\right)=f\cdot (exp\left[-b{g}^{T}Dg \right])+(1-f)\cdot exp\left[-b{D}_{water}\right]$$

where ${A}_{g}$ is the modeled attenuated signal (normalized by B0) for the applied diffusion gradient *g*, and *b* is the b-value (1000 s/mm^2^). The first term reflects the tissue compartment, where *D* is the diffusion tensor of this compartment, *f* is the fractional volume of the compartment, and *g*^*T*^ is the transpose of the vector *g*. The second term reflects an isotropic free-water compartment with a fractional volume of (1 − *f*), where the diffusion coefficient ${D}_{water}$ is set to the diffusivity of water at body temperature (3 × 10^−3^ mm^2^/s). AxD, RD, and FA were obtained from *D*. Standard DTI metrics are reported as AxD, RD, and FA, while FW-corrected DTI metrics are reported as AxD_t_, RD_t_, and FA_t_, where the *t* is indicative of greater tissue specificity from FW-DTI.

While FA indicates deviation from a spherical-shaped tensor (FA = 0; isotropic diffusion), the mode of anisotropy (MA) is a complementary measure that discriminates between linear and planar anisotropy. MA is mathematically orthogonal to FA and relates to the skewness of the DTI eigenvalues (${\lambda }_{1},{ \lambda }_{2},{ \lambda }_{3})$^37^:

2 $$MA=\frac{(-{\lambda }_{1}-{\lambda }_{2}+2{\lambda }_{3})(2{\lambda }_{1}-{\lambda }_{2}-{\lambda }_{3})(-{\lambda }_{1}+2{\lambda }_{2}-{\lambda }_{3})}{{2({\lambda }_{1}^{2}+{\lambda }_{2}^{2}+{\lambda }_{3}^{2}-{\lambda }_{1}{\lambda }_{2}-{\lambda }_{1}{\lambda }_{3}-{\lambda }_{2}{\lambda }_{3})}^{3/2}}$$

After quantifying all DTI-derived metrics, the B0 images from each subject were used to create a B0 group-wise template image using *buildtemplateparallel.sh* included in ANTs; this procedure has been demonstrated to improve the final normalization procedure^38^. The B0 group-wise template image was subsequently normalized to the IIT_mean_b0 image by ANTs symmetric image normalization (SyN) algorithm^39^. The warp fields obtained from this normalization and from the B0 group-wise template creation were used to transfer all DTI maps (standard and FW-corrected) to the Illinois Institute of Technology (IIT) Human Brain Atlas (v.4.1), which contains both anatomical and DTI brain templates in International Consortium for Brain Mapping (ICBM)-152 space^40^. All DTI maps in standard space were smoothed using FSL with an isotropic Gaussian kernel (sigma, 3 mm).

### Statistical analysis

Age, MMSE, and CDR scores are presented as mean and standard deviation (SD) for each group. Differences in age, sex, and cognitive test scores between groups were assessed using the Student's t-test. Comparisons of all DTI-, FW-DTI- metrics, and FW maps between groups were performed using the FSL Randomise tool^41^ with threshold‐free cluster enhancement (TFCE)^42^ and a correction for multiple comparisons via Family‐Wise Error (FWE) rate^38^ at a 0.01 level. A WM mask was defined using a threshold of 0.2 on the average values of FA (from standard DTI) and was applied to the other DTI maps. For each voxel, the DTI-derived metrics were fitted by an Analysis of Covariance model (ANCOVA), which included age and sex as covariates. The number of randomized permutations was set at 5,000. The clusters identified to be statistically different between groups were labeled according to the JHU DTI-based white-matter atlases^43,44^. All results are reported with a significance threshold of *p* value < 0.01, corrected for FWE, except where otherwise noted.

For the cognitively impaired group (AD only), the voxel-based relationship between MMSE scores and DTI metrics was assessed through Randomise with significance at *p*-value < 0.01 (FWE-corrected).

## Results

Student’s t-test did not show any difference in age (t = − 1.51; *p* = 0.137) or sex (t = 0.233; *p* = 0.817) between HC and AD. Significant differences were found for MMSE (t = − 5.21; *p* < 0.0001) and for CDR (t = − 8.22; *p* < 0.0001), as shown in Table 1.

### Standard DTI

**Figure 1:**
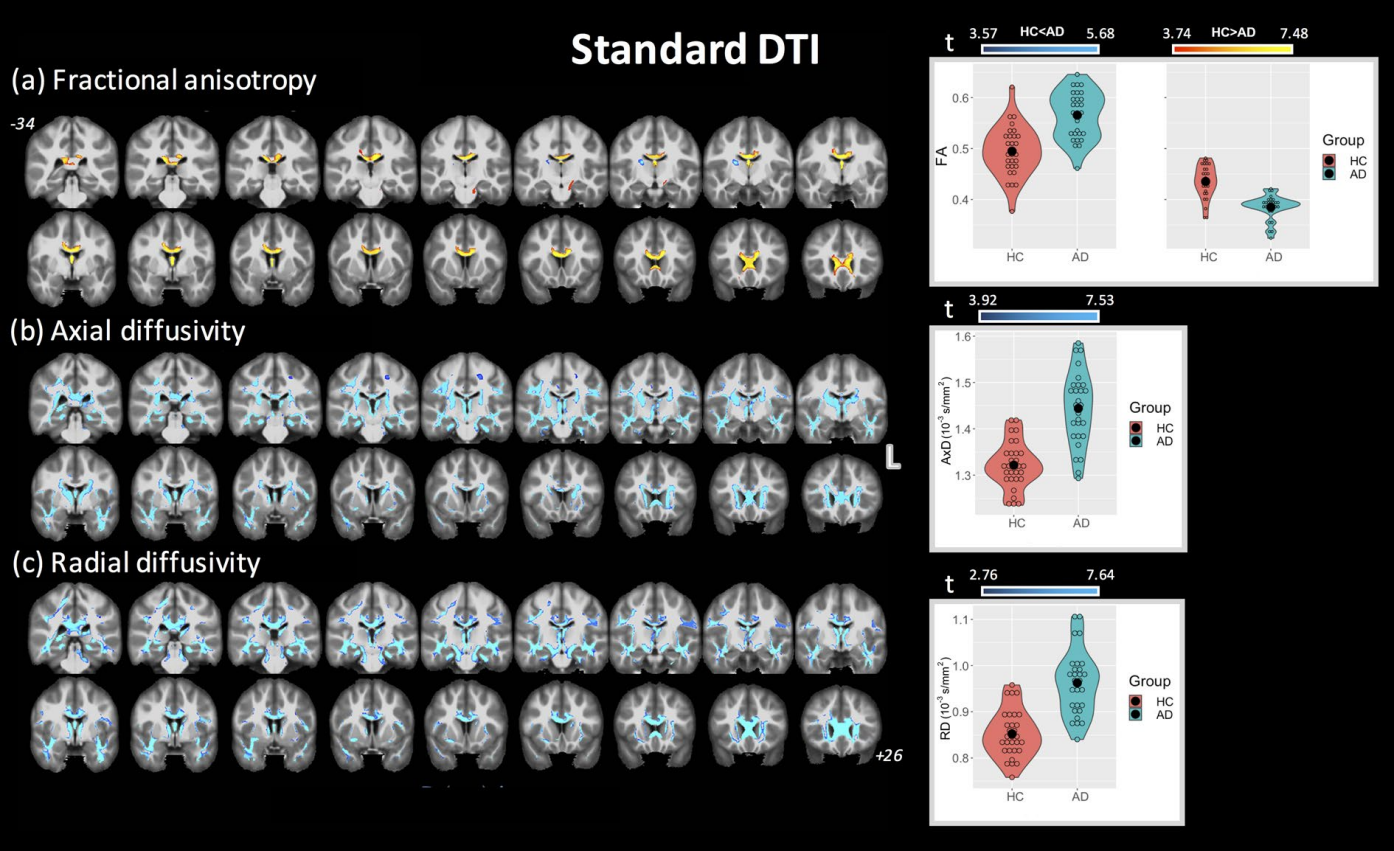
Coronal view of ANCOVA analysis between HC and AD subjects for standard DTI metrics. Clusters are reported at a significance threshold of *p*-value < 0.01 corrected for FWE. (**a**) FA, (**b**) AxD, and (**c**) RD analysis. The color bars indicate values that are lower in AD than HC (red-to-yellow) and higher in AD than HC (blue-to-cyan). For axial and radial diffusivities, only clusters with higher values in AD than HC were found. The corresponding violin plots represent the DTI metrics inside the significant clusters for each group.

Figure 1 shows the clusters of significant differences between the two groups obtained with standard DTI metrics with the corresponding violin plots quantifying the DTI differences for each group (across all combined clusters). For FA (panel (a)), lower FA was observed in AD compared with HC mainly in the fornix (cluster covering 93.3% of the fornix) and CC (covering 35.5% of CC). Small clusters where AD had higher FA values than HC were found in the right anterior thalamic radiation (ATR), cortical spinal tract (CST), posterior limb of internal capsule (PLIC), and superior corona radiata (SCR). More widespread differences between HC and AD were observed using AxD and RD (panel (b) and (c), respectively). Additionally, all significant clusters corresponded to higher values of AxD and RD metrics in the AD group, with no clusters corresponding to lower AxD or RD.

For each brain region, the significant cluster sizes (as percent volume) are reported in Table 2. Several clusters were observed where multiple DTI metrics were simultaneously altered between HC and AD groups, as shown in Supplementary Table 1. Regions where lower FA values overlapped with higher AxD and RD included the fornix, CC, forceps minor, and left cingulum. Higher FA values were found to overlap with higher AxD in the right PLIC, CST, and SCR. Substantial overlap was observed for clusters with higher AxD and higher RD, including 85% of the cingulum (hippocampus), 76% of the fornix, and 49% of the sagittal stratum. Finally, Supplementary Table 2 shows the corresponding mean DTI values inside each significant cluster for regions with higher and lower values for each group.

**Table 2:** Brain locations where clusters of significant differences between groups were found for both standard and FW-DTI analyses.

	Standard DTI				Free-water DTI					
	FA (HC) > FA (AD)	FA (HC) < FA (AD)	AD (HC) < AD (AD)	RD (HC) < RD (AD)	FA (HC) > FA (AD)	FA (HC) < FA (AD)	AD (HC) > AD (AD)	AD (HC) < AD (AD)	RD (HC) > RD (AD)	RD (HC) < RD (AD)*
	%	%	%	%	%	%	%	%	%	%
**JHU WM tract. atlas**										
Left ATR	–	–	34.13	31.06	–	7.91	0.42	1.95	6.12	–
Right ATR	–	1.14	31.99	28.51	–	9.96	0.53	1.64	6.79	–
Left CST	–	–	12.67	10.72	–	3.96	–	2.92	5.11	–
Right CST	–	1.59	14.34	16.38	–	8.47	–	6.06	8.97	–
Left CGC	6.41	–	9.77	38.37	1.90	–	4.34	–	–	–
Right CGC	–	–	24.44	41.83	–	–	1.62	–	–	–
CGH	–	–	60.13	61.44	–	–	–	–	3.8 (only R)	–
Forceps major	–	–	7.68	39.35	–	–	–	–	–	–
Forceps minor	7.76	–	18.98	33.10	4.78	–	5.60	–	–	–
IFOF left ATR	–	–	19.25	43.47	–	–	2.19	–	1.67	–
ILF	–	–	23.83	43.79	–	–	1.48	–	3.86	–
SLF	–	–	14.15	19.48	–	–	–	–	–	–
UF	–	–	30.18	48.35	–	–	0.61	–	–	–
**ICBM-DTI-81 WM atlas**										
Genu of CC	44.91	–	39.39	88.39	30.35	–	34.10	–	–	–
Body of CC	57.12	–	11.17	70.68	31.19	–	13.59	–	–	1.21
Splenium of CC	9.73	–	33.36	58.75	1.45	–	–	–	–	–
Fornix	93.33	–	73.93	93.74	70.91	–	45.99	–	–	–
Left SCP	–	–	–	10.60	–	–	–	–	–	–
CP	–	–	25.01	59.53	–	–	–	–	–	–
Anterior LIC	–	–	30.96	–	–	29.10	–	2.63	12.43	–
Right posterior LIC	–	5.83	39.59	–	–	39.77	–	30.58	38.67	–
Left posterior LIC	–	–	23.00	–	–	26.97	–	4.17	32.23	–
Retrolenticular part of IC	–	–	23.24	27.28	–	10.26	16.94	7.12	12.96	–
Right anterior CR	–	–	18.73	29.55	–	–	1.47	–	–	–
Left anterior CR	8.88	–	26.05	42.95	–	–	2.96	1.26	–	–
Right superior CR	–	1.22	28.58	–	–	17.71	–	6.93	18.13	–
Left superior CR	–	–	20.72	–	–	9.08	–	–	–	–
Right posterior CR	5.30	–	29.71	16.34	–	7.08	–	3.69	11.16	–
Left Posterior CR	–	–	14.94	–	–	–	–	2.98	–	–
Sagittal stratum	–	–	50.70	82.00	–	–	8.15	–	16.92 (only R)	–
External capsule	–	–	22.84	33.57	–	8.23	–	–	–	–
Right CGC	–	–	30.58	62.75	–	–	–	–	–	–
Left CGC	8.11	–	13.79	61.95	–	–	2.74	–	–	–
Cingulum (hippocampus)	–	–	85.22	86.79	–	–	–	–	–	–
SFOF	–	–	87.14	–	–	61.03	–	10.94	40.13	–

All results are shown for *p* < 0.01 (FWE corrected), except for the last column, indicated by *, where RD (HC) < RD (AD) is reported at *p* < 0.05 (FWE corrected). % columns indicate the percent volume of the cluster inside the corresponding brain area.

For JHU white-matter tractography atlas: *ATR* anterior thalamic radiation, *CST* cortical spinal tract, *CGC* cingulum (cingulate gyrus part), *CGH* cingulum (hippocampal part), *IFOF* inferior fronto-occipital fasciculus, *ILF* inferior longitudinal fasciculus, *UF* uncinate fasciculus. For ICBM-DTI-81 white-matter atlas: *CC* corpus callosum, *LIC* limb of internal capsule, *IC* internal capsule, *CR* corona radiate, *SS* sagittal stratum, *SFOF* superior fronto-occipital fasciculus.

### FW-DTI

**Figure 2:**
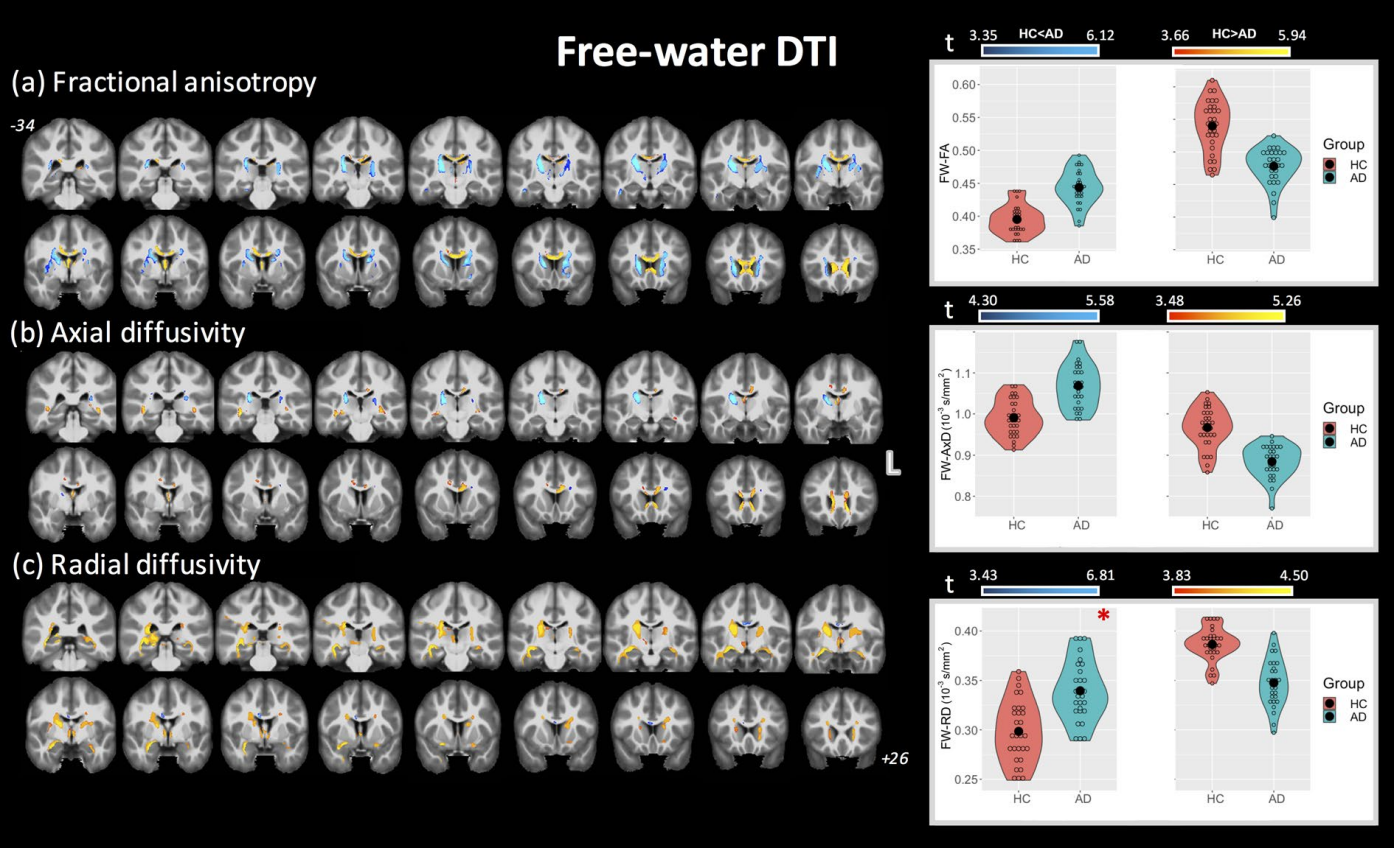
Coronal view of ANCOVA analysis between HC and AD subjects for the FW-DTI metrics. Clusters are reported at a significance threshold of *p*-value < 0.01 corrected for FWE, except for RD_t_ (HC) < RD_t_ (AD), which is reported at *p*-value < 0.05 FWE- corrected (**c**) and is indicated by the *. (**a**) FA_t_, (**b**) AxD_t_, and (**c**) RD_t_ analysis. The color bars indicate values that are lower in AD than HC (red-to-yellow) and higher in AD than HC (blue-to-cyan). The corresponding violin plots represent the FW-DTI metrics inside the significant clusters for each group.

Figure 2 shows the clusters of significant differences between groups for FW-DTI metrics with the corresponding violin plots quantifying the DTI differences for each group (across all combined clusters), where FA_t_, AxD_t_, and RD_t_ are displayed in panels (a), (b), and (c) (at *p* < 0.01 FWE corrected, unless otherwise indicated). The clusters where AD had lower FA_t_ values than HC correspond to the same WM locations as those observed using standard FA (specifically, fornix and CC); however, these clusters (covering 71% and 19.2% of fornix and CC, respectively) are smaller than standard FA (93.3% and 35.5%, respectively). Higher FA_t_ values in AD compared with HC were found bilaterally for ATR, CST, limb of internal capsule (LIC), SCR, and superior fronto-occipital fasciculus (SFOF). With regard to AxD_t_ and RD_t_, both higher and lower values were observed in multiple regions in the AD group (recall that only higher AxD and RD were observed using standard DTI). More specifically, AxD_t_ was lower in the AD group in CC and fornix and predominantly higher in the ATR, CST, and retrolenticular part of internal capsule. Lower RD_t_ was observed with AD mainly in the ATR, CST, PLIC, and SFOF, corresponding to similar areas with higher FA_t_. A small cluster inside the body of the CC showed higher RD_t_ in the AD group, albeit at *p* < 0.05 FWE corrected.

For each brain region, the significant cluster sizes (as percent volume) are reported in Table 2. As for standard DTI, several clusters were observed where multiple DTI metrics were simultaneously altered between HC and AD groups, as shown in Supplementary Table 1. No regions were observed where lower FA_t_ values overlapped with higher AxD_t_ and RD_t_; however, lower FA_t_ values overlapped with lower AxD_t_ and RD_t_ values in the fornix (4.8%), while lower FA_t_ corresponded with lower AxD_t_ in several regions including the CC and forceps minor. Higher FA_t_ values were found to overlap with higher AxD_t_ and lower RD_t_ in the SFOF, right PLIC, SCR, and anterior LIC. Several clusters were observed with higher AxD_t_ and lower RD_t_, including the left CST, right PLIC, and right SCR. Unlike standard DTI, no clusters demonstrated simultaneously higher AxD_t_ and higher RD_t_. Finally, Supplementary Table 3 shows the corresponding mean FW-DTI values inside each significant cluster for regions with higher and lower values for each group.

### FW-index

**Figure 3:**
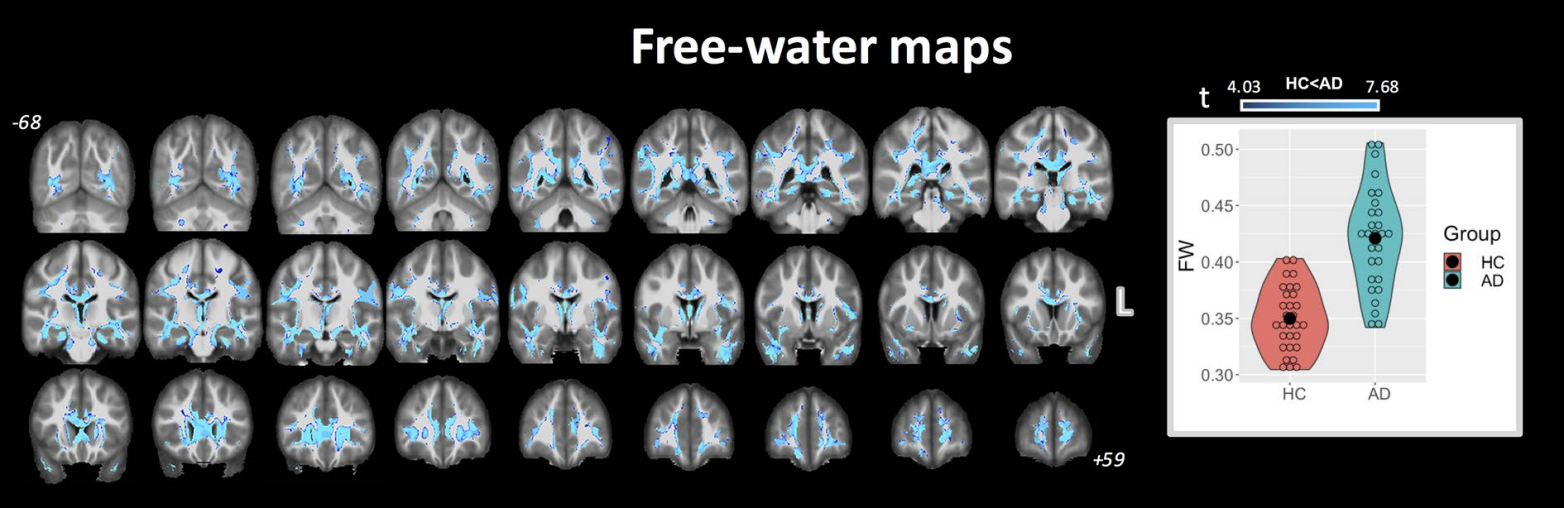
Coronal view of ANCOVA analysis between HC and AD subjects for FW index. Clusters are reported at a significance threshold of *p*-value < 0.01 corrected for FWE. Higher FW in AD compared to HC was observed widespread across cerebral WM. No significant clusters were found with FW (HC) > FW (AD). The corresponding violin plots represent the FW metrics inside the significant clusters for each group.

Figure 3 shows the significant differences between groups using the FW index (obtained from the fit in Eq. 1) at *p* < 0.01 FWE corrected. The corresponding violin plots are also shown for FW values for each group (across all combined clusters). Only clusters with higher FW values in AD compared with HC were found, with clusters covering 34% of the total WM mask. These differences were observed in several WM regions, including most prominently the fornix, cingulum (both hippocampal and cingulate gyrus parts), and CC. The complete list of group-wise significant clusters for the FW index between HC and AD is reported in Table 3.

**Table 3:** Brain locations where clusters of significant differences between groups were found for FW maps analysis at *p* < 0.01 (FWE corrected).

	%	FW (HC)	FW (AD)
**JHU WM tract. atlas**			
ATR	31.39	0.36 (0.03)	0.42 (0.04)
CST	12.98	0.30 (0.02)	0.35 (0.02)
CGC	56.34	0.28 (0.03)	0.33 (0.04)
CGH	85.16	0.33 (0.03)	0.41 (0.06)
Forceps major	40.10	0.33 (0.03)	0.38 (0.05)
Forceps minor	39.19	0.34 (0.04)	0.40 (0.04)
IFOF	39.92	0.28 (0.03)	0.32 (0.04)
ILF	52.05	0.27 (0.03)	0.32 (0.05)
SLF	30.08	0.28 (0.03)	0.33 (0.04)
UF	47.81	0.28 (0.03)	0.34 (0.04)
**ICBM-DTI-81 WM atlas**			
Genu of CC	67.27	0.39 (0.03)	0.45 (0.04)
Body of CC	47.89	0.37 (0.04)	0.43 (0.03)
Splenium of CC	52.17	0.30 (0.04)	0.36 (0.04)
Fornix	95.62	0.72 (0.06)	0.81 (0.05)
CP	41.40	0.33 (0.03)	0.38 (0.03)
Left anterior LIC	10.09	0.25 (0.03)	0.30 (0.04)
Right retrolenticular part of IC	21.22	0.28 (0.03)	0.32 (0.03)
Anterior CR	29.23	0.25 (0.04)	0.30 (0.04)
Right posterior CR	19.87	0.26 (0.03)	0.31 (0.04)
SS	65.31	0.29 (0.02)	0.34 (0.04)
EC	30.24	0.26 (0.03)	0.31 (0.04)
CGH	90.64	0.34 (0.03)	0.41 (0.06)

% columns indicate the percent volume of the cluster inside the corresponding brain area. Only clusters with higher FW values in AD than HC were observed.

For JHU white-matter tractography atlas: *ATR* anterior thalamic radiation, *CST* cortical spinal tract, *CGC* cingulum (cingulate gyrus part), *CGH* cingulum (hippocampal part), *IFOF* inferior fronto-occipital fasciculus, *ILF* inferior longitudinal fasciculus, *SLF* superior longitudinal fasciculus, *UF* uncinate fasciculus. For ICBM-DTI-81 white-matter atlas: *CC* corpus callosum, *CP* cerebral peduncle, *LIC* limb of internal capsule, *IC* internal capsule, *CR* corona radiata, *SS* sagittal stratum, *EC* external capsule.

### MA-index

**Figure 4:**
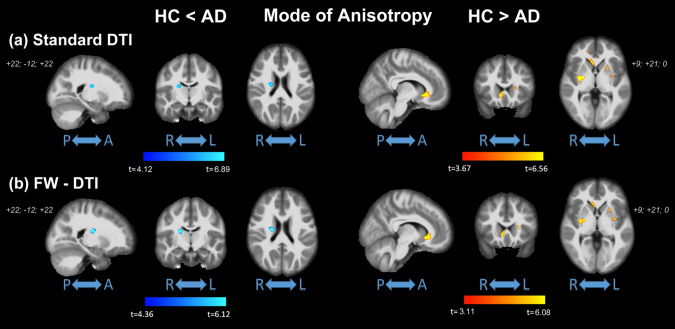
Coronal view of ANCOVA analysis between HC and AD subjects for MA index from (**a**) standard DTI and (**b**) FW-DTI. Left: MA in HC < MA in AD; right: MA in HC > MA in AD. Clusters are reported at a significance threshold of *p*-value < 0.01 corrected for FWE.

The results for MA are reported in Fig. 4. Significantly higher values of MA (from both standard DTI and FW-DTI) in AD compared with HC were observed in the right CST, right PLIC, right SCR, and right SFOF, corresponding to more linear anisotropy. On the other hand, lower MA values (from standard DTI and FW-DTI) in AD compared with HC were found mainly in the genu of CC and in the external capsule (EC), corresponding to more planar anisotropy. It should be noted that similar results for MA were obtained with both standard DTI and FW-DTI analysis. The complete list of group-wise significant clusters is reported in Table 4.

**Table 4:** Brain locations where clusters of significant differences between groups were found for MA index analysis at *p* < 0.01 (FWE corrected).

	Standard MA		FW-MA	
	HC < AD	HC > AD	HC < AD	HC > AD
	%	%	%	%
**JHU WM tract. atlas**				
Left ATR	–	0.87	–	0.50
Right CST	0.52	–	0.76	–
Forceps major	–	0.42	–	–
Forceps minor	–	1.73	–	0.87
IFOF	–	1.12	–	0.60
Left UF	–	0.51	–	–
Right UF	–	3.31	–	1.97
**ICBM-DTI-81 WM atlas**				
Genu of CC	–	10.07	–	6.76
Left anterior LIC	–	4.24	–	2.61
Right posterior LIC	1.61	–	2.81	–
Right anterior CR	–	1.29	–	–
Left anterior CR	–	1.70	–	0.68
Right superior CR	2.34	–	2.87	–
EC	–	10.40	–	6.26

% columns indicate the percent volume of the cluster inside the corresponding brain area.

For JHU white-matter tractography atlas: *ATR* anterior thalamic radiation, *CST* cortical spinal tract, *IFOF* inferior fronto-occipital fasciculus, *ILF* inferior longitudinal fasciculus, *UF* uncinate fasciculus. For ICBM-DTI-81 white-matter atlas: *CC* corpus callosum, *LIC* limb of internal capsule, *CR* corona radiata, *EC* external capsule, *SFOF* superior fronto-occipital fasciculus.

### Voxel-based correlations between DTI/FW-DTI metrics and MMSE

No significant voxel-based correlation was found between MMSE and either standard or FW-DTI metrics at *p* < 0.05 FWE corrected level.

### Correlations between standard and FW-DTI metrics

Supplementary Fig. 1 shows the population scatter-plots for standard and FW-DTI metrics, while the group-wise correlations for HC and AD are provided in each plot. Panels (a) and (b) show the correlations between FA and AxD, while panels (c) and (d) show the correlations between FA and RD. It should be noted that the correlations increased after FW correction (e.g., for the AD group: the correlations between FA and AxD increased from 0.160 (standard) to 0.856 (FW-corrected) and from − 0.464 (standard) to − 0.803 (FW-corrected) between FA and RD). Similar increases were observed for both HC and AD groups, suggesting that older HC participants also have substantial FW contributions to their dMRI-derived metrics.

Panels (e)–(g) show the correlations between FA and FW-FA_t_, AxD and FW-AxD_t_, RD and FW-RD_t_, respectively. By removing the isotropic diffusion contribution, the impact of FW-correction is to increase FA_t_, decrease AxD_t_, and decrease RD_t_, relative to the corresponding standard DTI metrics. These panels also show that FA is less affected by the FW component than AxD and RD (i.e., the correlation for standard vs. FW FA is higher than that for AxD and RD). As AxD and RD metrics are more influenced by free-water components (i.e., have lower correlations between standard and FW metrics), this is consistent with the widespread AxD and RD changes without FW-correction. Here again, there is not a strong group difference in the correlations. Additionally, the striking differences for AxD and RD metrics between standard and FW-correction can be attributed to their biophysical origin; that is, raw diffusivity parameters AxD and RD are likely to be more impacted by intravoxel free-water diffusion than the composite metric FA.

## Discussion

In this study, WM integrity was assessed in patients with Alzheimer’s disease using both standard and FW-corrected DTI metrics. Although DTI is a commonly used method for assessing WM, PVEs can reduce the accuracy of DTI metrics, ultimately limiting their interpretation. To mitigate PVE, free water‐contaminated voxels were removed by incorporating a FW component in the conventional diffusion modeling pipeline^18^, producing more accurate FW-corrected DTI metrics and the complementary FW index. Using both standard and FW-DTI, significant WM differences were observed between HC and AD subjects in several regions, including both higher and lower FA. Both AxD and RD were highly impacted by the effects of FW, and FW-corrected AxDt and RDt were more consistent with pathology-based expected spatial patterns of neurodegeneration. The FW index was higher in AD than HC across many regions, as expected. The FW correction method has shown promise in many neurological diseases of the WM^21,24–27^, particularly those with neurodegenerative aspects; similarly, we now demonstrate that the application of FW correction in AD improves the accuracy and sensitivity of the resulting DTI metrics.

Lower FA values, indicative of more isotropic motion, are consistent with neurodegenerative changes and were observed in AD in multiple regions, including most prominently the fornix and CC. Of particular interest, the fornix is the major output tract of the hippocampus and plays a major role in episodic memory^2,45^. In the context of AD, pathological changes in the fornix may occur at an early disease stage before clinical manifestations. Lower FA and higher MD in the fornix has been a robust and consistent finding in AD^46,47^ and may correlate with cognitive decline^46,48^. Similarly, FA reductions in the fornix have been identified using skeletonized tract-based spatial statistics (TBSS) in early-symptomatic individuals and those at high risk for developing AD^49–52^. However, the fornix is particularly prone to PVEs due to its proximity to the lateral ventricles^2^, which may be further exacerbated by atrophy-related ventricular expansion in AD. While decreases in standard FA may reflect a complex combination of atrophy and microstructural changes, these decreases remained after correction for FW, indicating that they reflect true pathological changes in the fornix.

Other significant changes were observed in AD in the CC, which is a major fiber bundle responsible for interhemispheric connection. Using both standard and FW-DTI, large clusters of lower FA were observed in the genu and body with minimal splenial changes, particularly using FW-DTI. Several other studies have similarly reported decreased FA in the CC; however, these studies have shown inconsistent regional changes with AD, with some studies finding changes throughout the CC^53^ and others reporting predominant changes in the genu^9^ or splenium^54^. Early involvement of the genu would support the anterior-to-posterior progression of the retrogenesis hypothesis, wherein degeneration occurs in reverse of myelination. In contrast, early changes in the splenium would be consistent the posterior-to-anterior progression of Wallerian degeneration following neuronal loss, wherein WM damage is subsequent to grey matter damage. To date, conclusive evidence of a gradient pattern has remained elusive^55,56^. Given the substantial FW component observed in the CC, the inclusion of FW correction algorithms could improve accuracy in the resulting DTI metrics in the presence of neurodegenerative changes, and future studies should consider whether the implementation of FW correction could increase confidence in the spatiotemporal patterns observed across DTI studies.

Higher FA_t_ in AD compared with HC was observed seemingly paradoxically using FW-DTI in bilateral ATR and CST, as well as internal capsule and corona radiata; with standard DTI, much smaller clusters of higher FA were observed in the right hemisphere (versus bilateral for FW-DTI) in the ATR, CST, PLIC and SCR (see Table 2 and Supplementary Tables 2 and 3). In the presence of a unidirectional fiber tract, higher FA would suggest lower RD and/or higher AxD, which are in contrast to the expected changes in the presence of neurodegeneration. However, one of the major drawbacks of standard DTI methods is an inability to resolve different fiber orientations within a voxel^57^, which arises from the assumption of Gaussian diffusion implicit in the tensor model^58^. In the presence of crossing fibers, FA is artificially diminished due to multi-directional fiber tracts; thus, AD-related neurodegeneration in these regions may yield increased FA due to the loss of crossing fibers. In these cases, MA—a metric of diffusion anisotropy—may provide insight in areas of crossing fibers; moreover, higher MA has been related to a loss of crossing fibers in early AD pathology in regions with known complicated tract topologies^59^. Further corroborating the presence of fiber crossing, MA was higher in AD subjects in the right CST, PLIC, and SCR. Conversely, lower MA in AD was found in clusters in the left ATR, forceps major and minor, IFOF, UF, genu of CC, anterior CR, and EC. Similar to these results, other studies have found parallel increases of both FA and MA in AD patients in a large cluster extending along the CST and involving the ATR^54,60^. The higher MA in AD subjects, together with higher FA, suggests a more linear shape of the diffusion tensor, indicative of the loss of crossing fiber populations in these areas. As the CST is relatively preserved in AD pathology, these changes suggest degeneration of fiber tracts in the ATR, CC, and fasciculus.

Using standard DTI, non-specific higher AxD and RD were observed across WM, while FW-AxD_t_ and FW-RD_t_ had better agreement with regional FA_t_ changes. This may be reflective of a larger impact of isotropic FW diffusion on AxD and RD metrics compared to FA. For example, lower FA in the CC and fornix is indicative of altered WM integrity (e.g., demyelination) in the AD group; these changes were driven by both higher AxD and RD with standard DTI, while FW correction showed that these changes in FA_t_ may be driven by underlying lower AxD_t_. Using FW-DTI, lower FW-AxD_t_ (AD < HC) were found in WM locations corresponding to lower FA_t_, while the fornix showed simultaneous lower FW-RD_t_ (HC > AD) in a small cluster. With standard DTI, AxD was higher across multiple regions, consistent with prior literature^14,49,54,61,62^. However, the mechanism of higher AxD is unknown and contrary to prevailing theories of known AD pathology^61^. Using FW-DTI, regions of lower AxD_t_ corresponded to lower FA_t_ values, suggesting that prior reports of higher AxD may have been sensitive to the effects of FW. A similar trend of AxD decreasing with FW correction was previously reported using TBSS in the context of AD and cerebrovascular disease^21^. After FW correction, higher AxD_t_ in AD, compared with HCs, was observed in regions with known crossing fibers, consistent with degeneration of select fiber tracts in these regions. Additionally, clusters with higher FW-FA_t_ values in AD (e.g., ATR, CST, anterior/posterior LIC, and SFOF) were associated with higher FW-AxD_t_ and lower FW-RD_t_. Using standard DTI analysis, no lower AxD or RD values were detected in AD subjects.

The FW volume fraction was quantified using the FW-correction algorithm and, consistent with AD-related neurodegeneration, was higher in AD in many WM areas and tracts. More specifically, large clusters of higher FW were located in the cingulum (hippocampal and cingulate gyrus parts, 85.16% and 56.34%, respectively), ILF and IFOF (52% and 40%, respectively), forceps major and minor (approximately 40% each), and ATR (31.39%). Similar clusters were also found in the fornix (95.6%) and CC (genu, body, and splenium; approximately 56% of the total CC). Additionally, approximately 42% of the FW clusters were located in WM outside the regions specified by the atlases; in particular, FW increases were found in several regions bordering various sulcal fundi. Consistent with DTI-based metrics, the FW index further demonstrates an important connection between the fornix, CC, and AD. The high volume of FW in these regions may be related to loss of WM integrity; moreover, the contaminating effects of FW further validates the significant differences observed between standard and FW-DTI metrics. This metric may itself be a valuable biomarker, consistent with our hypothesis, as previous studies have shown that higher FW values were associated with poorer executive functioning, visual construction, and visuomotor performance^21^. Overall, FW-DTI provides not only more accurate and consistent DTI-based biomarkers, but also provides quantitative FW volume fractions, which may be a potential biomarker of sub-voxel neurodegenerative processes.

Correlations between DTI metrics and various neuropsychology assessments could lend insight into the role of WM changes on cognitive function in AD pathology. In this study, we did not observe any significant voxel-wise correlations between MMSE and DTI metrics before or after FW correction in the AD cohort. This may be attributable to the similar group characteristics (predominantly very mild to mild AD) and the relatively narrow range of MMSE scores, while future studies could consider including more sensitive measures of cognitive functioning, including various sub-domains such as memory and executive function. Despite the lack of correlation in this study, the overall finding that FW-DTI metrics are more consistent with the pathology may lend insight into the inconsistent correlative findings across the literature. While the relationship between WM integrity as assessed by DTI and cognitive functions remains unsettled, the implementation of FW correction to yield more accurate DTI metrics should be considered as a methodological improvement for future correlation studies, particularly for the diffusivity metrics AxD and RD.

There are several limitations to this study. First, we used a voxel-based approach for WM analysis rather than a skeletonized approach, such as TBSS. As TBSS minimizes tract variance across subjects using a common skeletonized tract, it uses fewer voxels and is inherently less sensitive to partial volume effects. However, the higher number of voxels available with voxel-based approaches may increase sensitivity to subtle differences in microstructure^63^. Even so, all of these approaches necessitate the use of atlases, which may be limited in the WM regions that are represented (in this case, the atlases covered 84% of the WM mask); this is particularly relevant for the FW index that showed widespread changes across WM, even outside of the atlas regions. In the present study, atrophy likely minimal due to the selected patient population, further enabling the use of a voxel-based approach. Second, no significant voxel-based correlations (*p* < 0.01 FWE corrected) were observed between standard DTI and FW-DTI metrics and the MMSE score, which is consistent with several previous studies^13–16^. This suggests that while WM is altered in AD, the relationship between global cognitive scores and WM pathology may not be linear. Future work could consider more sensitive measures of cognitive subdomains, and the inclusion of a longitudinal aspect may further pinpoint the temporal ordering of WM alterations relative to GM pathology and cognitive decline. Finally, as early biomarkers are of increasing interest, future studies should also include cohorts of MCI or subjects at high risk of developing AD.

## Conclusion

In conclusion, this study demonstrates that the implementation of a FW correction algorithm for DTI improves both the sensitivity and specificity of derived DTI metrics by removing PVEs, and better captures underlying AD-related pathologic changes than standard DTI approaches. We found significantly altered DTI metrics in AD compared to HC for both standard and FW-DTI, but importantly FW-DTI identified changes were more consistent with known AD pathology as hypothesized (both in terms of magnitude and direction of DTI changes). In addition, MA may improve specificity related to crossing fibers, while the FW index may improve sensitivity to sub-voxel neurodegeneration. Overall, FW-DTI improves the reliability and inter-parameter consistency of DTI metrics in the presence of atrophy, and the resulting metrics provide more sensitive and specific insight into AD-related pathological changes in white matter.

## Supplementary Information

Supplementary Information.

## References

[1] Ferri CP, Prince M, Brayne C, Brodaty H, Fratiglioni L, Ganguli M (2005). Global prevalence of dementia: A Delphi consensus study. Lancet.

[2] Oishi K, Lyketsos CG (2014). Alzheimer's disease and the fornix. Front. Aging Neurosci..

[3] Sexton CE, Kalu UG, Filippini N, Mackay CE, Ebmeier KP (2011). A meta-analysis of diffusion tensor imaging in mild cognitive impairment and Alzheimer's disease. Neurobiol. Aging.

[4] Jones DK, Cercignani M (2010). Twenty-five pitfalls in the analysis of diffusion MRI data. NMR Biomed..

[5] Vos SB, Jones DK, Jeurissen B, Viergever MA, Leemans A (2012). The influence of complex white matter architecture on the mean diffusivity in diffusion tensor MRI of the human brain. Neuroimage.

[6] Concha L (2014). A macroscopic view of microstructure: Using diffusion-weighted images to infer damage, repair, and plasticity of white matter. Neuroscience.

[7] Liu Y, Spulber G, Lehtimaki KK, Kononen M, Hallikainen I, Grohn H (2011). Diffusion tensor imaging and tract-based spatial statistics in Alzheimer's disease and mild cognitive impairment. Neurobiol. Aging.

[8] Stricker NH, Schweinsburg BC, Delano-Wood L, Wierenga CE, Bangen KJ, Haaland KY (2009). Decreased white matter integrity in late-myelinating fiber pathways in Alzheimer's disease supports retrogenesis. Neuroimage.

[9] Ukmar M, Makuc E, Onor ML, Garbin G, Trevisiol M, Cova MA (2008). Evaluation of white matter damage in patients with Alzheimer's disease and in patients with mild cognitive impairment by using diffusion tensor imaging. Radiol. Med..

[10] Bozzali M, Falini A, Franceschi M, Cercignani M, Zuffi M, Scotti G (2002). White matter damage in Alzheimer's disease assessed in vivo using diffusion tensor magnetic resonance imaging. J. Neurol. Neurosurg. Psychiatry.

[11] Shim G, Choi KY, Kim D, Suh SI, Lee S, Jeong HG (2017). Predicting neurocognitive function with hippocampal volumes and DTI metrics in patients with Alzheimer's dementia and mild cognitive impairment. Brain Behav..

[12] Xue Y, Zhang Z, Wen C, Liu H, Wang S, Li J (2019). Characterization of Alzheimer's disease using ultra-high b-values apparent diffusion coefficient and diffusion kurtosis imaging. Aging Dis..

[13] Ibrahim I, Horacek J, Bartos A, Hajek M, Ripova D, Brunovsky M (2009). Combination of voxel based morphometry and diffusion tensor imaging in patients with Alzheimer's disease. Neuro Endocrinol. Lett..

[14] Mayo CD, Garcia-Barrera MA, Mazerolle EL, Ritchie LJ, Fisk JD, Gawryluk JR (2018). Relationship between DTI metrics and cognitive function in Alzheimer's disease. Front. Aging Neurosci..

[15] Patil RB, Ramakrishnan S (2014). Analysis of sub-anatomic diffusion tensor imaging indices in white matter regions of Alzheimer with MMSE score. Comput Methods Programs Biomed.

[16] Stahl R, Dietrich O, Teipel SJ, Hampel H, Reiser MF, Schoenberg SO (2007). White matter damage in Alzheimer disease and mild cognitive impairment: Assessment with diffusion-tensor MR imaging and parallel imaging techniques. Radiology.

[17] Pierpaoli C, Jezzard P, Basser PJ, Barnett A, Di Chiro G (1996). Diffusion tensor MR imaging of the human brain. Radiology.

[18] Pasternak O, Sochen N, Gur Y, Intrator N, Assaf Y (2009). Free water elimination and mapping from diffusion MRI. Magn. Reson. Med..

[19] Metzler-Baddeley C, O'Sullivan MJ, Bells S, Pasternak O, Jones DK (2012). How and how not to correct for CSF-contamination in diffusion MRI. Neuroimage.

[20] Hoy AR, Ly M, Carlsson CM, Okonkwo OC, Zetterberg H, Blennow K (2017). Microstructural white matter alterations in preclinical Alzheimer's disease detected using free water elimination diffusion tensor imaging. PLoS ONE.

[21] Ji F, Pasternak O, Liu S, Loke YM, Choo BL, Hilal S (2017). Distinct white matter microstructural abnormalities and extracellular water increases relate to cognitive impairment in Alzheimer's disease with and without cerebrovascular disease. Alzheimers Res. Ther..

[22] Fletcher E, Carmichael O, Pasternak O, Maier-Hein KH, DeCarli C (2014). Early brain loss in circuits affected by alzheimer's disease is predicted by fornix microstructure but may be independent of gray matter. Front. Aging Neurosci..

[23] Maier-Hein KH, Westin CF, Shenton ME, Weiner MW, Raj A, Thomann P (2015). Widespread white matter degeneration preceding the onset of dementia. Alzheimers Dement..

[24] Ofori E, DeKosky ST, Febo M, Colon-Perez L, Chakrabarty P, Duara R (2019). Free-water imaging of the hippocampus is a sensitive marker of Alzheimer's disease. Neuroimage Clin..

[25] Arribarat G., O. Pasternak, A. De Barros, M. Galitzky, O. Rascol and P. Peran, Substantia nigra locations of iron-content, free-water and mean diffusivity abnormalities in moderate stage Parkinson's disease. *Parkinsonism Relat. Disord.* **65**, 146–152 (2019). 10.1016/j.parkreldis.2019.05.033. 10.1016/j.parkreldis.2019.05.033 31182373

[26] Bergamino M, Kuplicki R, Victor TA, Cha YH, Paulus MP (2017). Comparison of two different analysis approaches for DTI free-water corrected and uncorrected maps in the study of white matter microstructural integrity in individuals with depression. Hum. Brain Mapp..

[27] Bergamino M, Pasternak O, Farmer M, Shenton ME, Hamilton JP (2016). Applying a free-water correction to diffusion imaging data uncovers stress-related neural pathology in depression. Neuroimage Clin..

[28] Dumont M, Roy M, Jodoin PM, Morency FC, Houde JC, Xie Z (2019). Free water in white matter differentiates MCI and AD from control subjects. Front. Aging Neurosci..

[29] Marcus DS, Fotenos AF, Csernansky JG, Morris JC, Buckner RL (2010). Open access series of imaging studies: Longitudinal MRI data in nondemented and demented older adults. J. Cogn. Neurosci..

[30] Ardekani BA, Bachman AH, Figarsky K, Sidtis JJ (2014). Corpus callosum shape changes in early Alzheimer's disease: An MRI study using the OASIS brain database. Brain Struct. Funct..

[31] Bachman AH, Lee SH, Sidtis JJ, Ardekani BA (2014). Corpus callosum shape and size changes in early Alzheimer's disease: A longitudinal MRI study using the OASIS brain database. J. Alzheimers Dis..

[32] Folstein MF, Folstein SE, McHugh PR (1975). "Mini-mental state". A practical method for grading the cognitive state of patients for the clinician. J. Psychiatr. Res..

[33] Khan, T.K. Clinical Diagnosis of Alzheimer’s Disease in Biomarkers in Alzheimer's Disease, Elsevier, Editor. (Academic Press, 2016). ISBN 978-0-12-804832-0. 10.1016/C2015-0-04217-4.

[34] Smith SM, Jenkinson M, Woolrich MW, Beckmann CF, Behrens TE, Johansen-Berg H (2004). Advances in functional and structural MR image analysis and implementation as FSL. Neuroimage.

[35] Andersson JLR, Sotiropoulos SN (2016). An integrated approach to correction for off-resonance effects and subject movement in diffusion MR imaging. Neuroimage.

[36] Smith SM (2002). Fast robust automated brain extraction. Hum. Brain Mapp..

[37] Ennis DB, Kindlmann G (2006). Orthogonal tensor invariants and the analysis of diffusion tensor magnetic resonance images. Magn. Reson. Med..

[38] Keihaninejad S, Ryan NS, Malone IB, Modat M, Cash D, Ridgway GR (2012). The importance of group-wise registration in tract based spatial statistics study of neurodegeneration: A simulation study in Alzheimer's disease. PLoS ONE.

[39] Avants BB, Epstein CL, Grossman M, Gee JC (2008). Symmetric diffeomorphic image registration with cross-correlation: Evaluating automated labeling of elderly and neurodegenerative brain. Med. Image Anal..

[40] Varentsova A, Zhang S, Arfanakis K (2014). Development of a high angular resolution diffusion imaging human brain template. Neuroimage.

[41] Winkler AM, Ridgway GR, Webster MA, Smith SM, Nichols TE (2014). Permutation inference for the general linear model. Neuroimage.

[42] Smith SM, Nichols TE (2009). Threshold-free cluster enhancement: Addressing problems of smoothing, threshold dependence and localisation in cluster inference. Neuroimage.

[43] Hua K, Zhang J, Wakana S, Jiang H, Li X, Reich DS (2008). Tract probability maps in stereotaxic spaces: Analyses of white matter anatomy and tract-specific quantification. Neuroimage.

[44] Wakana S, Caprihan A, Panzenboeck MM, Fallon JH, Perry M, Gollub RL (2007). Reproducibility of quantitative tractography methods applied to cerebral white matter. Neuroimage.

[45] Hopper MW, Vogel FS (1976). The limbic system in Alzheimer's disease. A neuropathologic investigation. Am. J. Pathol..

[46] Oishi K, Mielke MM, Albert M, Lyketsos CG, Mori S (2012). The fornix sign: A potential sign for Alzheimer's disease based on diffusion tensor imaging. J. Neuroimaging.

[47] Ringman JM, O'Neill J, Geschwind D, Medina L, Apostolova LG, Rodriguez Y (2007). Diffusion tensor imaging in preclinical and presymptomatic carriers of familial Alzheimer's disease mutations. Brain.

[48] Mielke MM, Kozauer NA, Chan KC, George M, Toroney J, Zerrate M (2009). Regionally-specific diffusion tensor imaging in mild cognitive impairment and Alzheimer's disease. Neuroimage.

[49] Bosch B, Arenaza-Urquijo EM, Rami L, Sala-Llonch R, Junque C, Sole-Padulles C (2012). Multiple DTI index analysis in normal aging, amnestic MCI and AD. Relationship with neuropsychological performance. Neurobiol. Aging.

[50] Damoiseaux JS, Smith SM, Witter MP, Sanz-Arigita EJ, Barkhof F, Scheltens P (2009). White matter tract integrity in aging and Alzheimer's disease. Hum. Brain Mapp..

[51] Douaud G, Menke RA, Gass A, Monsch AU, Rao A, Whitcher B (2013). Brain microstructure reveals early abnormalities more than two years prior to clinical progression from mild cognitive impairment to Alzheimer's disease. J. Neurosci..

[52] Honea RA, Vidoni E, Harsha A, Burns JM (2009). Impact of APOE on the healthy aging brain: A voxel-based MRI and DTI study. J. Alzheimers Dis..

[53] Sun X, Salat D, Upchurch K, Deason R, Kowall N, Budson A (2014). Destruction of white matter integrity in patients with mild cognitive impairment and Alzheimer disease. J. Investig. Med..

[54] Palesi F, De Rinaldis A, Vitali P, Castellazzi G, Casiraghi L, Germani G (2018). Specific patterns of white matter alterations help distinguishing Alzheimer's and vascular dementia. Front. Neurosci..

[55] Alves GS, Knochel VO, Knochel C, Carvalho AF, Pantel J, Engelhardt E (2015). Integrating retrogenesis theory to Alzheimer's disease pathology: Insight from DTI-TBSS investigation of the white matter microstructural integrity. Biomed. Res. Int..

[56] Di Paola M, Di Iulio F, Cherubini A, Blundo C, Casini AR, Sancesario G (2010). When, where, and how the corpus callosum changes in MCI and AD: A multimodal MRI study. Neurology.

[57] Tuch DS, Reese TG, Wiegell MR, Makris N, Belliveau JW, Wedeen VJ (2002). High angular resolution diffusion imaging reveals intravoxel white matter fiber heterogeneity. Magn. Reson. Med..

[58] Alexander AL, Hasan KM, Lazar M, Tsuruda JS, Parker DL (2001). Analysis of partial volume effects in diffusion-tensor MRI. Magn. Reson. Med..

[59] Douaud G, Jbabdi S, Behrens TE, Menke RA, Gass A, Monsch AU (2011). DTI measures in crossing-fibre areas: Increased diffusion anisotropy reveals early white matter alteration in MCI and mild Alzheimer's disease. Neuroimage.

[60] Teipel SJ, Grothe MJ, Filippi M, Fellgiebel A, Dyrba M, Frisoni GB (2014). Fractional anisotropy changes in Alzheimer's disease depend on the underlying fiber tract architecture: A multiparametric DTI study using joint independent component analysis. J. Alzheimers Dis..

[61] Acosta-Cabronero J, Alley S, Williams GB, Pengas G, Nestor PJ (2012). Diffusion tensor metrics as biomarkers in Alzheimer's disease. PLoS ONE.

[62] Huang H, Fan X, Weiner M, Martin-Cook K, Xiao G, Davis J (2012). Distinctive disruption patterns of white matter tracts in Alzheimer's disease with full diffusion tensor characterization. Neurobiol. Aging.

[63] Preti MG, Baglio F, Lagana MM, Griffanti L, Nemni R, Clerici M (2012). Assessing corpus callosum changes in Alzheimer's disease: Comparison between tract-based spatial statistics and atlas-based tractography. PLoS ONE.

