# Supplementary material for: Free-water diffusion tensor imaging improves the accuracy and sensitivity of white matter analysis in Alzheimer’s disease
Source: Sci Rep. 2021 Mar 26;11:6990. doi: 10.1038/s41598-021-86505-7 (PMC7998032; doi:10.1038/s41598-021-86505-7)

Supplementary Information for

Free-Water Diffusion Tensor Imaging improves the accuracy and sensitivity of white matter analysis in Alzheimer's disease

Maurizio Bergamino, Ryan R. Walsh, Ashley M. Stokes

For both standard and FW-DTI, several clusters were observed where multiple DTI metrics were simultaneously altered between HC and AD groups, as shown in Supplementary Table 1. For each atlas, the corresponding region names can be found in Table 2.

Supplementary Table 1: Volumes (%) of the overlapped clusters for standard and FW-DTI. Metrics with ↑ indicate HC < AD, while ↓ indicates HC > AD.

	Standard DTI					FW-DTI							
	FA ↓			FA ↑	AxD ↑ & RD ↑	FA_t ↓			FA_t ↑			AxD_t ↑ & RD_t ↓	AxD_t ↓ & RD_t ↓
	AxD ↑	RD ↑	AxD & RD ↑	AxD ↑		AxD_t ↓	RD_t ↓	AxD_t & RD_t ↓	AxD_t ↑	RD_t ↓	AxD_t ↑ & RD_t ↓		
**JHU WM tract. atlas**	% volume			% volume	% volume	% volume			% volume			% volume	% volume
Left ATR	-	-	-	-	21.45	-	-	-	-	3.75	-	-	-
Right ATR	-	-	-	-	22.74	-	-	-	-	-	-	-	-
Left CST	-	-	-	-	5.12	-	-	-	2.84	5.74	2.84	2.84	-
Right CST	-	-	-	1.59	3.77	-	-	-	-	-	-	-	-
Left CCG	-	6.36	-	-	9.00	1.77	-	-	-	-	-	-	-
Right CCG	-	-	-	-	24.26	-	-	-	-	-	-	-	-
CGH	-	-	-	-	59.12	-	-	-	-	-	-	-	-
Forceps_Major	-	-	-	-	7.17	-	-	-	-	-	-	-	-
Forceps_Minor	3.61	7.76	3.61	-	17.75	2.42	-	-	-	-	-	-	-
IFOF	-	-	-	-	16.73	-	-	-	-	-	-	-	0.41
ILF	-	-	-	-	20.15	-	-	-	-	-	-	-	-
SLF	-	-	-	-	11.22	-	-	-	-	-	-	-	-
UF	-	-	-	-	27.84	-	-	-	-	-	-	-	-
**ICBM-DTI-81 WM atlas**													
Genu of CC	21.05	44.88	21.05	-	39.63	17.80	-	-	-	-	-	-	-
Body of CC	9.59	52.54	9.59	-	11.79	7.90	-	-	-	-	-	-	-
Splenium of CC	2.86	9.18	2.86	-	26.18	-	-	-	-	-	-	-	-
Fornix	75.08	93.33	75.08	-	75.50	28.68	6.15	4.80	-	-	-	-	4.80
CP	-	-	-	-	26.47	-	-	-	-	-	-	-	-
Right Anterior limb of IC	-	-	-	-	-	-	-	-	1.87	12.36	1.87	1.87	-
Left Anterior limb of IC	-	-	-	-	-	-	-	-	-	9.45	-	-	-
Right Posterior limb of IC	-	-	-	5.83	-	-	-	-	14.94	30.22	14.76	14.76	-
Retrolenticular part of IC	-	-	-	-	7.54	-	-	-	4.02	7.35	2.73	3.02	0.19
Right Anterior CR	-	-	-	-	11.64	-	-	-	-	-	-	-	-
Left Anterior CR	-	8.71	-	-	14.98	0.70	-	-	-	-	-	-	-
Right Superior CR	-	-	-	1.22	-	-	-	-	5.40	16.52	5.40	5.40	-
Left Superior CR	-	-	-	-	-	-	-	-	-	4.62	-	-	-
Right Posterior CR	-	3.74	-	-	4.97	-	-	-	2.58	7.00	2.58	2.58	-
Posterior thalamic radiation	-	-	-	-	8.08	-	-	-	-	-	-	-	-
Sagittal stratum	-	-	-	-	49.32	-	-	-	-	-	-	-	3.11
External capsule	-	-	-	-	17.95	-	-	-	-	2.91	-	-	-
Right Cingulum	-	-	-	-	32.46	-	-	-	-	-	-	-	-
Left Cingulum	1.93	8.11	1.93	-	14.72	0.47	-	-	-	-	-	-	-
Cingulum (hippocampus)	-	-	-	-	84.60	-	-	-	-	-	-	-	-
SFOF	-	-	-	-	-	-	-	-	4.93	39.45	4.93	4.93	-

For standard DTI, significant differences in FA, AxD, and RD between HC and AD were observed in regions across the brain (shown in Figure 1 violin plots across all combined clusters). Supplementary Table 2 shows the mean DTI values inside each significant cluster for regions with higher and lower values for each group.

Supplementary Table 2: DTI values in brain regions where clusters were found between groups for standard DTI analysis at p<0.01 (FWE corrected). The percent volume corresponding to each cluster can be found in Table 2.

	FA (HC) > FA (AD)		FA (HC) < FA (AD)		AxD (HC) < AxD (AD)		RD (HC) < RD (AD)	
**JHU WM tract. atlas**	**FA (HC)**	**FA (AD)**	**FA (HC)**	**FA (AD)**	**AxD (HC)**	**AxD (AD)**	**RD (HC)**	**RD (AD)**
Left ATR	-	-	-	-	1.35 (0.072)	1.47 (0.078)	9.27 (0.59)	10.3 (0.69)
Right ATR	-	-	0.472 (0.058)	0.562 (0.057)	1.35 (0.069)	1.47 (0.091)	9.32 (0.64)	10.4 (0.80)
Left CST	-	-	-	-	1.41 (0.061)	1.51 (0.070)	9.16 (0.62)	10.4 (0.79)
Right CST	-	-	0.540 (0.063)	0.628 (0.056)	1.34 (0.062)	1.44 (0.073)	7.76 (0.44)	8.70 (0.53)
Left CGC	0.436 (0.043)	0.372 (0.043)	-	-	1.13 (0.051)	1.23 (0.072)	7.20 (0.53)	7.98 (0.55)
Right CGC	-	-	-	-	1.10 (0.047)	1.19 (0.067)	7.48 (0.49)	8.25 (0.54)
CGH	-	-	-	-	1.30 (0.074)	1.46 (0.129)	9.16 (0.60)	10.6 (1.13)
Forceps Major	-	-	-	-	1.69 (0.070)	1.81 (0.080)	8.02 (0.61)	9.06 (0.95)
Forceps Minor	0.514 (0.045)	0.457 (0.039)	-	-	1.40 (0.056)	1.51 (0.068)	8.12 (0.63)	9.15 (0.66)
IFOF Left ATR	-	-	-	-	1.21 (0.041)	1.30 (0.057)	7.33 (0.40)	8.11 (0.58)
ILF	-	-	-	-	1.19 (0.051)	1.30 (0.077)	7.40 (0.39)	8.27 (0.63)
SLF	-	-	-	-	1.12 (0.039)	1.20 (0.066)	7.54 (0.44)	8.30 (0.58)
UF	-	-	-	-	1.18 (0.039)	1.27 (0.057)	7.43 (0.43)	8.18 (0.55)
**ICBM-DTI-81 WM atlas**	**FA (HC)**	**FA (AD)**	**FA (HC)**	**FA (AD)**	**AxD (HC)**	**AxD (AD)**	**RD (HC)**	**RD (AD)**
Genu of CC	0.515 (0.046)	0.456 (0.040)	-	-	1.88 (0.077)	2.01 (0.087)	8.84 (0.86)	10.2 (0.82)
Body of CC	0.539 (0.043)	0.476 (0.036)	-	-	1.93 (0.104)	2.06 (0.090)	8.65 (0.86)	9.77 (0.65)
Splenium of CC	0.596 (0.037)	0.547 (0.033)	-	-	1.77 (0.090)	1.91 (0.077)	7.19 (0.79)	8.30 (0.76)
Fornix	0.348 (0.044)	0.274 (0.042)	-	-	2.61 (0.201)	2.89 (0.194)	18.1 (1.12)	21.1 (1.80)
Left SCP	-	-	-	-	-	-	9.11 (0.87)	9.91 (0.94)
CP	-	-	-	-	1.78 (0.074)	1.91 (0.120)	8.31 (0.60)	9.40 (0.76)
Anterior LIC	-	-	-	-	1.26 (0.071)	1.36 (0.089)	-	-
Right Posterior LIC	-	-	0.494 (0.054)	0.578 (0.054)	1.20 (0.054)	1.28 (0.068)	-	-
Left Posterior LIC	-	-	-	-	1.20 (0.061)	1.27 (0.069)	-	-
Retrolenticular part of IC	-	-	-	-	1.42 (0.075)	1.52 (0.076)	7.47 (0.53)	8.21 (0.68)
Right Anterior CR	-	-	-	-	1.18 (0.058)	1.26 (0.066)	7.29 (0.64)	7.99 (0.59)
Left Anterior CR	0.355 (0.039)	0.307 (0.036)	-	-	1.16 (0.052)	1.24 (0.071)	7.18 (0.53)	7.89 (0.60)
Right Superior CR	-	-	0.526 (0.071)	0.620 (0.061)	1.18 (0.072)	1.27 (0.084)	-	-
Left Superior CR	-	-	-	-	1.18 (0.085)	1.28 (0.099)	-	-
Right Posterior CR	0.413 (0.060)	0.351 (0.061)	-	-	1.37 (0.086)	1.48 (0.085)	7.17 (0.53)	7.93 (0.89)
Left Posterior CR	-	-	-	-	1.38 (0.089)	1.48 (0.097)	-	-
Sagittal stratum	-	-	-	-	1.41 (0.064)	1.56 (0.113)	7.14 (0.38)	8.19 (0.85)
External capsule	-	-	-	-	1.18 (0.045)	1.27 (0.093)	7.34 (0.39)	8.13 (0.71)
Right CGC	-	-	-	-	1.11 (0.058)	1.20 (0.078)	7.55 (0.51)	8.33 (0.59)
Left CGC	0.421 (0.034)	0.360 (0.043)	-	-	1.16 (0.060)	1.26 (0.094)	7.32 (0.59)	8.20 (0.70)
Cingulum (Hippocampus)	-	-	-	-	-	-	-	-
SFOF	-	-	-	-	1.18 (0.069)	1.28 (0.101)	-	-

Additionally, for FW-DTI, significant differences in FA_t, AxD_t, and RD_t between HC and AD were observed in regions across the brain (shown in Figure 2 violin plots across all combined clusters). Supplementary Table 3 shows the mean DTI values inside each significant cluster for regions with higher and lower values for each group.

Supplementary Table 3: FW-DTI values in brain regions where clusters were found between groups for FW-DTI analysis at $p<0.01$ (FWE corrected). The percent volume corresponding to each cluster can be found in Table 2.

	FA_t (HC) > FA_t (AD)		FA_t (HC) < FA_t (AD)		AxD_t (HC) > AxD_t (AD)		AxD_t (HC) < AxD_t (AD)		RD_t (HC) > RD_t (AD)		RD_t (HC) < RD_t (AD)*	
**JHU WM tract. atlas**	**FA_t (HC)**	**FA_t (AD)**	**FA_t (HC)**	**FA_t (AD)**	**AxD_t (HC)**	**AxD_t (AD)**	**AxD_t (HC)**	**AxD_t (AD)**	**RD_t (HC)**	**RD_t (AD)**	**RD_t (HC)**	**RD_t (AD)**
ATR	-	-	0.429 (0.057)	0.508 (0.052)	0.96 (0.021)	0.91 (0.037)	0.92 (0.034)	0.99 (0.054)	3.99 (0.020)	3.64 (0.023)	-	-
CST	-	-	0.665 (0.037)	0.714 (0.041)	-	-	1.06 (0.044)	1.12 (0.053)	1.06 (0.044)	1.12 (0.053)	-	-
Left CGC	0.575 (0.064)	0.494 (0.073)	-	-	0.93 (0.054)	0.88 (0.051)	-	-	-	-	-	-
Right CGC	-	-	-	-	0.84 (0.048)	0.80 (0.041)	-	-	-	-	-	-
Right CGH	-	-	-	-	-	-	-	-	4.18 (0.023)	3.80 (0.040)	-	-
Forceps Minor	0.697 (0.045)	0.629 (0.047)	-	-	1.00 (0.074)	0.92 (0.052)	-	-	-	-	-	-
Left IFOF	-	-	-	-	1.03 (0.023)	0.97 (0.034)	-	-	-	-	-	-
Right IFOF	-	-	-	-	1.05 (0.032)	0.98 (0.046)	-	-	3.55 (0.020)	3.15 (0.030)	-	-
Left ILF	-	-	-	-	1.05 (0.021)	1.01 (0.026)	-	-	-	-	-	-
Right ILF	-	-	-	-	1.04 (0.032)	0.98 (0.044)	-	-	4.25 (0.019)	3.87 (0.034)	-	-
UF	-	-	-	-	1.02 (0.054)	0.96 (0.053)	-	-	-	-	-	-
**ICBM-DTI-81 WM atlas**	**FA_t (HC)**	**FA_t (AD)**	**FA_t (HC)**	**FA_t (AD)**	**AxD_t (HC)**	**AxD_t (AD)**	**AxD_t (HC)**	**AxD_t (AD)**	**RD_t (HC)**	**RD_t (AD)**	**RD_t (HC)**	**RD_t (AD)**
Genu of CC	0.682 (0.047)	0.612 (0.047)	-	-	1.00 (0.076)	0.91 (0.052)	-	-	-	-	-	-
Body of CC	0.704 (0.042)	0.640 (0.036)	-	-	1.03 (0.052)	0.96 (0.045)	-	-	-	-	2.92 (0.030)	3.31 (0.031)
Splenium of CC	0.793 (0.029)	0.752 (0.026)	-	-	-	-	-	-	-	-	-	-
Fornix	0.581 (0.084)	0.464 (0.104)	-	-	0.86 (0.014)	0.69 (0.013)	-	-	-	-	-	-
Right Anterior LIC	-	-	0.479 (0.070)	0.561 (0.070)	-	-	1.02 (0.056)	1.10 (0.071)	3.65 (0.024)	3.33 (0.023)	-	-
Left Anterior LIC	-	-	0.453 (0.075)	0.537 (0.056)	-	-	-	-	4.09 (0.026)	3.77 (0.026)	-	-
Posterior LIC	-	-	0.581 (0.044)	0.644 (0.043)	-	-	1.02 (0.046)	1.09 (0.055)	3.59 (0.025)	3.26 (0.022)	-	-
Retrolenticular part of IC	-	-	0.661 (0.049)	0.714 (0.035)	1.06 (0.029)	1.00 (0.044)	0.95 (0.047)	1.02 (0.053)	3.44 (0.018)	3.22 (0.022)	-	-
Right Anterior CR	-	-	-	-	0.99 (0.064)	0.93 (0.047)	-	-	-	-	-	-
Left Anterior CR	-	-	-	-	0.97 (0.043)	0.92 (0.042)	0.87 (0.035)	0.93 (0.051)	-	-	-	-
Right Superior CR	-	-	0.560 (0.065)	0.642 (0.071)	-	-	1.02 (0.078)	1.12 (0.083)	3.77 (0.031)	3.39 (0.034)	-	-
Left Superior CR	-	-	0.510 (0.067)	0.595 (0.078)	-	-	-	-	-	-	-	-
Right Posterior CR	-	-	0.656 (0.058)	0.716 (0.039)	-	-	0.98 (0.070)	1.07 (0.069)	3.25 (0.024)	2.94 (0.026)	-	-
Left Posterior CR	-	-	-	-	-	-	0.97 (0.050)	1.05 (0.061)	-	-	-	-
Right SS	-	-	-	-	1.04 (0.043)	0.97 (0.065)	-	-	3.65 (0.025)	3.19 (0.040)	-	-
Left SS	-	-	-	-	1.01 (0.039)	0.94 (0.054)	-	-	-	-	-	-
External capsule	-	-	0.448 (0.044)	0.499 (0.027)	-	-	-	-	-	-	-	-
Left CGC	-	-	-	-	0.92 (0.053)	0.87 (0.059)	-	-	-	-	-	-
SFOF	-	-	0.475 (0.052)	0.547 (0.079)	-	-	0.93 (0.054)	1.01 (0.071)	4.06 (0.025)	3.73 (0.035)	-	-

Finally, Supplementary Figure 1 shows the population scatterplots for standard and FW-DTI metrics, while the group-wise correlations for HC (red lines) and AD (green lines) are provided in each plot. The inter-parameter correlations between FA and both AxD (a,b) and RD (c,d) improve after FW correction (a vs. b and c vs. d). As expected, within each parameter, FW correction tends to increase FA and decrease both AxD and RD, with RD decreased more substantially. FA is less impacted by FW correction, as evidenced by the relatively high correlations (panel (e)). The correlations are similar between the HC and AD groups.

Supplementary Figure 1: (a) and (b) show the correlations between FA and AxD; panels (c) and (d) show the correlations between FA and RD; panels (e), (f), and (g) show the correlations between FA and FW-FA$_t$, AxD and FW-AxD$_t$, RD and FW-RD$_t$, respectively.

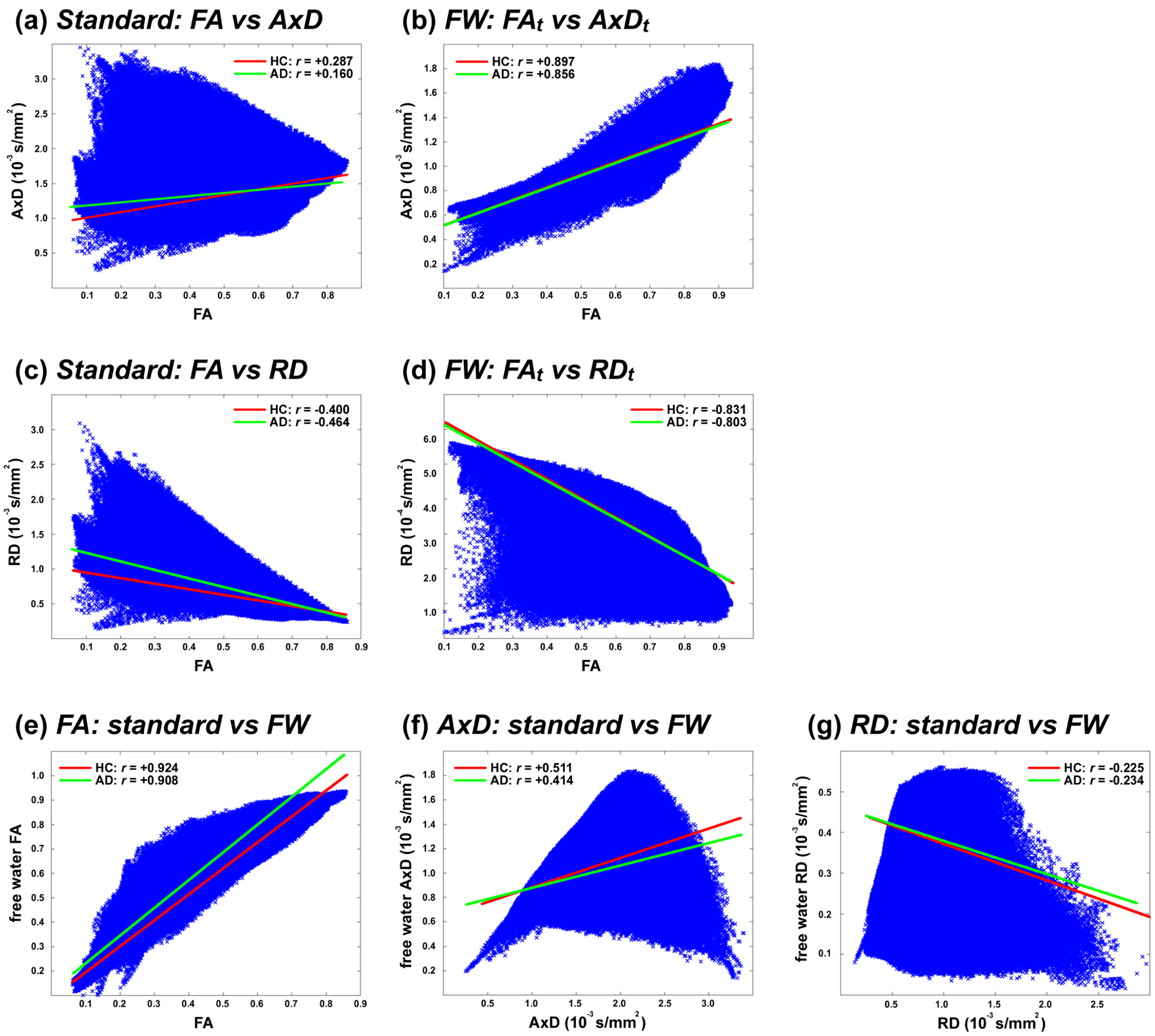

Supplement: Supplementary file 1 — Supplementary Information. [file 41598_2021_86505_MOESM1_ESM.pdf]
